# Comparative Transcriptome Profiling of Ovary Tissue between Black Muscovy Duck and White Muscovy Duck with High- and Low-Egg Production

**DOI:** 10.3390/genes12010057

**Published:** 2020-12-31

**Authors:** Xiuyu Bao, Yiping Song, Tao Li, Shanshan Zhang, Lihua Huang, Shuya Zhang, Junting Cao, Xiaolin Liu, Jianqin Zhang

**Affiliations:** College of Animal Science and Technology, Northwest A&F University, Yangling 712100, China; xiuyu@nwafu.edu.cn (X.B.); s1157903447@163.com (Y.S.); 18392459744@nwafu.edu.cn (T.L.); zhangSS2017051704@nwafu.edu.cn (S.Z.); 2017051700@nwafu.edu.cn (L.H.); shuyazhang2020@163.com (S.Z.); fightingcaoting@nwafu.edu.cn (J.C.); liuxiaolin@nwsuaf.edu.cn (X.L.)

**Keywords:** Muscovy duck, transcriptome, ovary, reproduction, gene expression

## Abstract

The egg-laying rate is an important indicator for evaluating fertility of poultry. In order to better understand the laying mechanism of Muscovy ducks, gene expression profiles and pathways of ovarian tissues in high- and low-laying black (BH and BL) and white Muscovy ducks (WH and WL) during the peak production period were performed by using RNA-seq. The total number of reads produced for each ovarian sample ranged from 44,344,070 to 47,963,328. A total of 113, 619 and 87 differentially expressed genes (DEGs) were identified in BH-vs-WH, BL-vs-BH and BL-vs-WL, respectively. Among them, 54, 356 and 49 genes were up regulated and 59, 263 and 38 genes were down regulated. In addition, there were only 10 up-regulated genes in WL-vs-WH. In the comparison of DEGs in black and white Muscovy ducks, two co-expressed DEG genes were detected between BH-vs-WH and BL-vs-WL and seven DEGs were co-expressed between BL-vs-BH and WL-vs-WH. The RNA-Seq data were confirmed to be reliable by qPCR. Numerous DEGs known to be involved in ovarian development were identified, including *TGFβ*2, *NGFR*, *CEBPD*, *CPEB*2, *POSTN*, *SMOC*1, *FGF*18, *EFNA*5 and *SDC*4. Gene Ontology (GO) annotations indicated that DEGs related to ovarian development were mainly enriched in biological processes of “circadian sleep/wake cycle process,” “negative regulation of transforming growth factor-β secretion,” “positive regulation of calcium ion transport” in BH-vs-WH and “cell surface receptor signaling pathway,” “Notch signaling pathway” and “calcium ion transport” in BL-vs-BH. Besides, “steroid biosynthetic process,” “granulosa cell development” and “egg coat formation” were mainly enriched in BL-vs-WL and “reproduction,” “MAPK cascade” and “mitotic cell cycle” were mainly enriched in WL-vs-WH. KEGG pathway analysis showed that the PI3K-Akt signaling pathway and ovarian steroidogenesis were the most enriched in Muscovy duck ovary transcriptome data. This work highlights potential genes and pathways that may affect ovarian development in Muscovy duck.

## 1. Introduction

Reproductive performance, an important economic trait of poultry, is mainly determined by the egg production. Improving egg production ability is an important breeding objective. With traditional breeding methods, the reproductive performance of laying duck has been gradually improved but more significant improvements have been slow [1]. Ovary is a female reproductive organ, which is closely related to egg-laying characteristics and the ovarian function of poultry directly affects the egg production [2]. Ovarian follicular development is a complex process, involving a variety of endocrine, autocrine and paracrine factors, which control the proliferation and differentiation of oocytes, granulosa cells and thecal cells [3]. The number of primordial follicle pools in the ovary is fixed and the quiescence, survival and activation of follicles in the pools depends on the dynamic balance, which is regulated by many signaling molecules or pathways [4,5]. Genetic or environmental factors affect follicular development, which may lead to follicular inactivation or premature activation and even degeneration and atresia at different stages of development [6]. Therefore, the regulation of ovarian function, including the identification and analysis of reproductive-related genes, has been concerned to improve the reproductive performance of poultry. For example, transforming growth factor-β family (TGF-β), fibroblast growth factors (FGFs), insulin-like growth factor family (IGFs) [7], *FSHR* [8], *GnRH* [9], *STAR* [10] and *PRLR* [11] have been shown to be associated with poultry ovarian function and affect egg production.

Although candidate genes for avian reproduction have been identified, the specific molecular mechanisms and related signaling pathways are poorly understood. As a next-generation sequencing technology, RNA-seq is used to explore the function of genes by detecting the gene expression in animal samples [12]. In recent years, the ovarian transcriptome of livestock, such as pig [13], cattle [14], sheep [15], has been studied by using RNA-seq. Similarly, there are many studies on the transcriptome of poultry ovary. Zhang et al. (2019) found that there were 305 differentially expressed genes (DEGs) identified in the ovaries of hens with relatively greater and lesser egg production. Besides, five candidate genes related to egg production, including *ZP*2, *WNT*4, *AMH*, *IGF*1 and *CYP17A*1 genes, were discovered [16]. Tao et al. (2017) performed a comparative transcriptomic analysis of the ovaries of Jinding ducks with high and low egg production using RNA-seq, the results showed that 367 of 843 DEGs were down regulated in high egg production ovaries and 476 DEGs were up-regulated in low egg production ovaries [17]. Luan et al. (2014) identified the DEGs in the ovaries of Huoyan geese between the laying period and the ceased period, 344 up- and 344 down-regulated genes were classified into functional categories and 12 DEGs that were mainly involved in pathways for reproduction regulation [18]. Xu et al. (2016) compared the gene expression of pigeon ovary at different stages, there were 409 DEGs between pre- and post-ovulation. However, there are few reports on the high- and low-yield ovarian transcriptome of Muscovy duck [19].

Muscovy duck, known for its strong adaptability, fertility and high meat yield [20], is native to tropical areas of Central and South America. In China, black Muscovy and white Muscovy are mainly raised. The egg-laying peak period of Muscovy duck is between 35 and 53 weeks, covering most of the egg-laying period [21]. Although these ducks have been raised on a large scale in China, the poor egg production performance and the lack of systematic breeding have resulted in uneven production performance in Muscovy duck and seriously affects the economic benefits of many enterprises. Under the same conditions of nutrition and feeding management, the egg production performance of Muscovy duck is mainly determined by genes and related regulatory factors. Therefore, the purpose of our research was to identify the DEGs and pathways and analyze the similarities and differences of the differential expression in the ovarian tissues of Muscovy ducks with high- and low-yield by using RNA-seq. Through a comprehensive analysis of gene expression levels reflecting the ovaries of black and white Muscovy ducks, the genes and molecular mechanisms involved in this process have been found. All results provide theoretical basis for the breeding of Muscovy ducks and are useful for understanding of the molecular regulation mechanism of egg production characteristics in black and white Muscovy duck.

## 2. Materials and Methods

### 2.1. Animal and Sample Collection

The egg production of Muscovy ducks aged 216 to 280 days (65 days in total) was recorded. More than 60 eggs were considered as high-laying black Muscovy duck (BH) and white Muscovy duck (WH) and 15–20 eggs were defined as low-yield duck (BL and WL). Six black Muscovy ducks (3 high-yield and 3 low-yield) and 6 white Muscovy ducks (half each for high- and low-yield laying ducks), raised under the same environmental conditions with free access to feed and water (Table 1), were randomly selected and slaughtered quickly to collect the ovarian tissues, which comprised the whole ovary including the small and large yellow follicles. The samples were immediately frozen in liquid nitrogen and stored at −80 °C until further use. Total RNA was extracted from ovarian tissue of Muscovy duck using TRIzol reagent according to the manufacturer’s instructions (Takara, Dalian, China). The purity and concentration of RNA were detected by agarose gel electrophoresis and Nanodrop 2000 (Thermo, Waltham, MA, USA) and RNA integrity was measured using the 2100 Bioanalyzer (Agilent Technologies, San Jose, CA, USA). All samples had an RNA integrity number (RIN) ≥ 7.8 and a RNA concentration ≥ 875.6 ng/μL (Table S1). The tested Muscovy ducks were purchased from Anda Farm in Tongguan County, Shaanxi Province, China and raised according to the standard procedure. Besides this, the Muscovy ducks were known to be unrelated to each other for at least three generations. All animal procedures were performed according to guidelines provided by the China Council on Animal Care and the protocols were approved by the Experimental Animal Management Committee (EAMC) of Northwest A&F University (ethic code: #0726/2018).

### 2.2. Library Construction and Sequencing

Twelve RNA samples with high quality (average RIN: 8.4 ± 0.251) and concentration (average concentration: 1136.9 ± 3.828 ng/μL) were used to construct the sequencing libraries and high-throughput sequencing was performed on the Illumina novaseq 6000 platform (San Diego, CA, USA) following the manufacturer’s recommendations, generating 150 bp paired-end reads. The high-quality clean reads were obtained by filtering the raw reads and removing: (1) the sequences containing adapters; (2) the sequences with more than 10% of N bases; (3) the sequences with more than 50% base quality values less than 10. The clean reads were aligned to the *Anas platyrhynchos* genome (https://www.ncbi.nlm.nih.gov/genome/?term=duck) and annotated transcripts (https://www.ncbi.nlm.nih.gov/assembly/GCF_003850225.1) by way of the Tophat 2 software (http://ccb.jhu.edu/software/hisat2/index.shtml).

### 2.3. Differential Expression Analysis

The number of clean reads for each gene was calculated and the Fragment Per Kilobases per Million Fragment (FPKM) was used to estimate the expression abundance of transcripts from different samples [22]. Differential expression analyses of the 4 comparison groups (BL-vs-BH, WL-vs-WH, BH-vs-WH and BL-vs-WL) were performed using the DESeq R package (http://www.bioconductor.org/packages/release/bioc/html/DESeq.html) [23,24] and genes with an *p*-value ≤ 0.05 and an expression |log2 Fold | ≥ 1were identified as DEGs.

### 2.4. GO and KEGG Analysis

To annotate the function of these DEGs, Gene Ontology (GO) analysis was conducted by using the GOseq software (https://bioconductor.org/packages/release/bioc/html/goseq.html) for each of the three main categories: biological process, cellular component and molecular function. Biological pathways enriched for the identified DEGs through KEGG pathway analyses were carried out using the KOBAS software (http://kobas.cbi.pku.edu.cn/kobas3/?t=1).

### 2.5. Verification of RNA-seq by qPCR

To verify the reliability of the RNA-Seq results, 4 DEGs (*TGFβ*2, *FGF*18, *POSTN*, *SMOC*1) in the ovary of black Muscovy duck and 3 DEGs (*POSTN*, *SMOC*1, *COL4A*1) in white Muscovy duck were randomly selected, respectively. The β-actin gene of duck was used as the control (NC_040060.1), after the stability of β-actin was tested in this system. The RNA extracted from same ovaries with an RIN ≥ 7.8, A260/280 >1.8 and A260/230 > 2.0 were used for cDNA synthesis according to the manufacturer’s instructions (Takara, Dalian, China). The primers information were listed in Table 2. qPCR was run in triplicate and performed in a EcoRT48 system (OSA, London, UK), with a 10 μL reaction volume containing 5 μL SYBR Premix Ex Taq II (Takara, Dalian, China), 0.8 µL cDNA (750 ng/µL), 0.2 µL of each primer and 3.8 µL RNase-free ddH_2_O. The qPCR program was 95 °C for 30 s; followed by 40 cycles of 95 °C for 5 s, 60 °C for 30 s and then 95 °C for 15 s, 55 °C for 15 s, 95 °C for 15 s. Gene expression results were calculated using the 2^−ΔΔCt^ method.

### 2.6. Statistical Analysis

Experiments were repeated 3 times and presented as mean ± standard deviation and conducted using one-way ANOVA with Dunnet’s t-test at *p* < 0.05 probability levels in SPSS18.0. “*” was considered significant difference (*p* < 0.05); “**” was considered extremely significant difference (*p* < 0.01).

## 3. Results

### 3.1. High-Throughput Sequencing and Read Mapping

In this study, a total of 12 libraries in high- and low-yield of ovarian tissue in Muscovy ducks were established by high-throughput RNA sequencing. The clean reads of each sample were more than 44 million, the mapped reads and unique mapped reads ranged from 60.28% to 67.40% and 57.81% to 64.36%, respectively. In addition, more than 16,276 genes were detected in each sample (Table 3)

### 3.2. Differentially Expressed Genes

**Figure 1:**
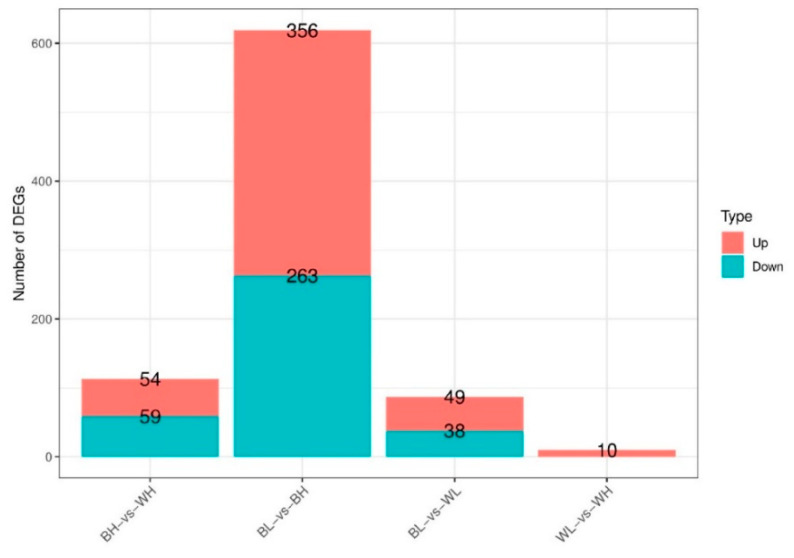
Statistic of differentially expressed genes (DEGs).

**Figure 2:**
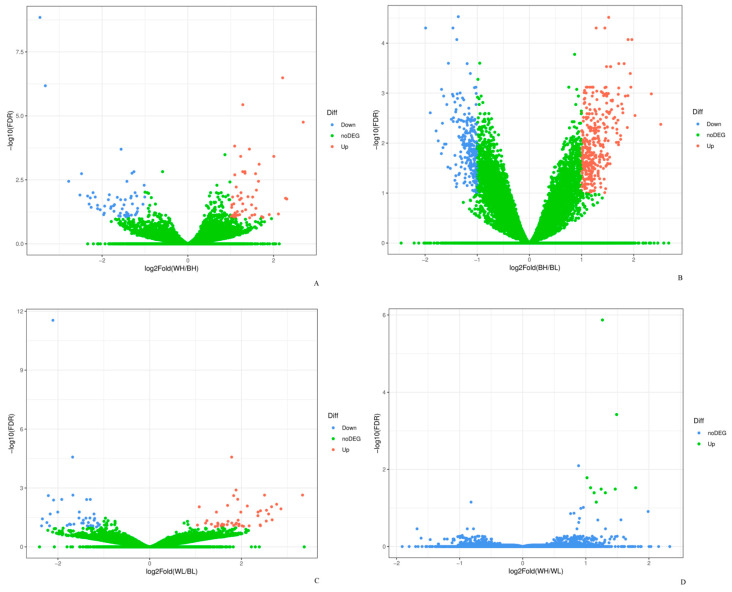
The Volcano plot of DEGs in (**A**) BH-vs-WH, (**B**) BL-vs-BH, (**C**) BL-vs-WL and (**D**) WL-vs-WH.

According to the correlation analysis of 12 samples, the differences between biological replicates were small and the repeatability was high, which indicated that the selection of experimental samples was consistent and reliable (Figure S1). A comparison of the gene expression showed that a total of 113 genes were differentially expressed between BH and WH, including 54 up-regulated genes and 59 down-regulated genes. 619 DEGs were discovered in BL-vs-BH and 356 DEGs were up-regulated genes and 263 DEGs were down-regulated genes. In BL-vs-WL, there were 87 DEGs, of which 49 were up-regulated and 38 were down-regulated genes. In addition, compared with WH, there were only 10 up-regulated genes in WL group (Figure 1 and Figure 2).

**Figure 3:**
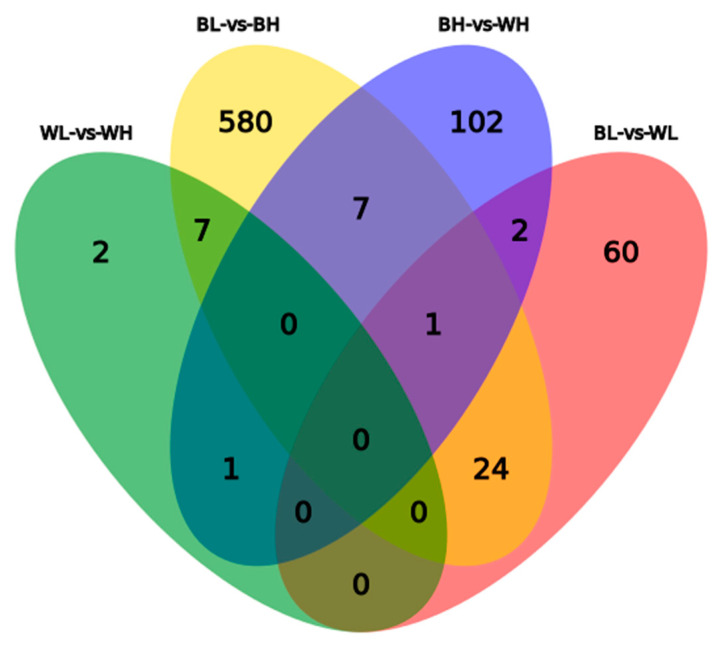
A Venn diagram of DEGs showing overlap between the four comparisons.

A comparative analysis of DEGs obtained by black and white Muscovy ducks showed that two co-expressed DEG genes were found between BH-vs-WH and BL-vs-WL and 7 co-expressed DEGs between BL-vs-BH and WL-vs-WH (Figure 3).

### 3.3. Gene Ontology and KEGG Analysis

**Figure 4:**
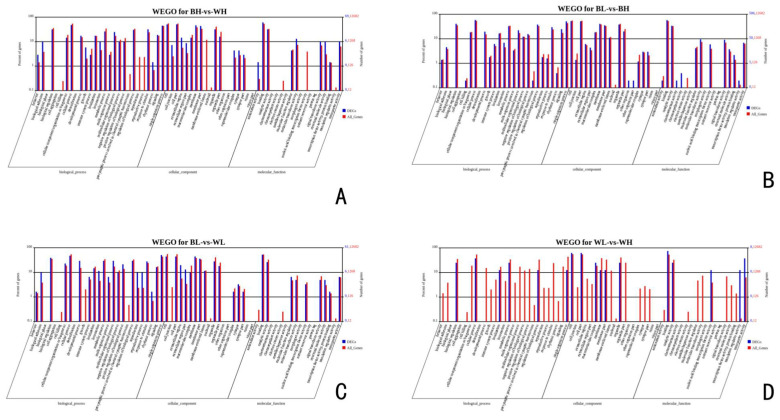
Gene Ontology (GO) terms for DEGs in (**A**) BH-vs-WH, (**B**) BL-vs-BH, (**C**) BL-vs-WL and (**D**) WL-vs-WH.

To further assess the functional roles of DEGs in ovarian development, a GO enrichment analysis was performed. The most significant biological processes related to ovarian development in BH-vs-WH, included “circadian sleep/wake cycle process,” “negative regulation of transforming growth factor-β secretion” and “positive regulation of calcium ion transport.” The most relevant terms for cellular component were “actin cytoskeleton” and “focal adhesion.” Significant GO terms for molecular function included “extracellular matrix structural constituent” and “calcium channel regulator activity.” In BL-vs-BH, DEGs related to ovarian development were mainly enriched in biological processes of “cell surface receptor signaling pathway,” “Notch signaling pathway” and “calcium ion transport.” “Voltage-gated calcium channel complex” and “proteasome core complex” were mainly enriched in cellular component and “calcium ion binding” and “low-density lipoprotein receptor activity” were mainly enriched in molecular function. The common DEGs related to ovarian development in BL-vs-WL were primarily enriched in biological processes of “steroid biosynthetic process,” “granulosa cell development” and “egg coat formation.” DEGs were primarily enriched in cellular component, including “voltage-gated calcium channel complex” and “cyclin-dependent protein kinase holoenzyme complex.” There were some terms mainly enriched in molecular function of “transforming growth factor β receptor binding” and “calcium ion binding.” Besides, several fundamental biological processes related to ovarian development were found to be notably enriched in WL-vs-WH, such as “reproduction,” “MAPK cascade” and “mitotic cell cycle” and several cellular component were found to be notably enriched, such as “cyclin-dependent protein kinase holoenzyme complex” and “proteasome complex.” Furthermore, DEGs were primarily enriched in molecular function, including “translation regulator activity” and “lipid kinase activity” (Figure 4).

**Figure 5:**
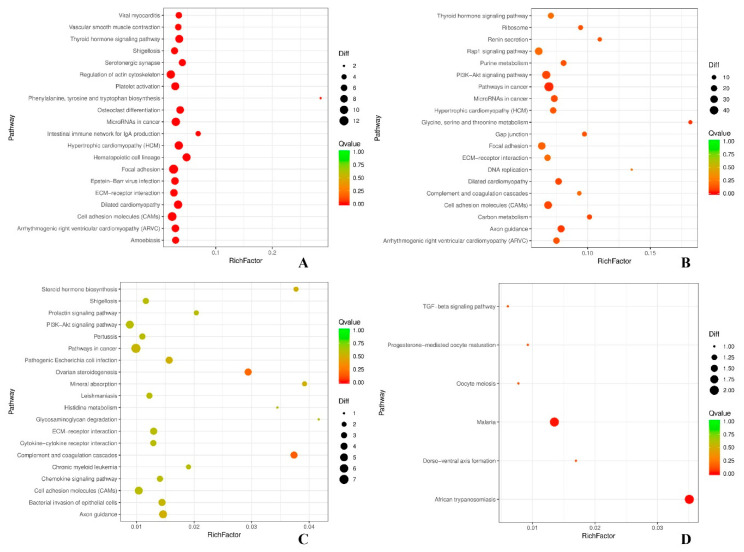
KEGG pathways for DEGs in (**A**) BH-vs-WH, (**B**) BL-vs-BH, (**C**) BL-vs-WL and (**D**) WL-vs-WH. Note: In the figure, each circle represents a KEGG pathway, the name of which being *Scheme 3*. 4. Verification of Differentially Expressed Genes.

To better understand the biological functions and interaction of genes, a KEGG pathway analysis was performed for the DEGs identified. In the first 20 pathways enriched by DEGs of the comparisons, the KEGG pathways enriched significantly (*P*-value < 0.05) were mainly concentrated in PI3K-Akt signaling pathway, Rap1 signaling pathway, cell adhesion molecules (CAMs), thyroid hormone signaling pathway, focal adhesion, ECM-receptor interaction, ovarian steroidogenesis, progesterone-mediated oocyte maturation, oocyte meiosis and TGF-β signaling pathway. Among them, PI3K-Akt signaling pathway and ovarian steroidogenesis were involved in the reproductive process of black and white Muscovy ducks (Figure 5).

**Figure 6:**
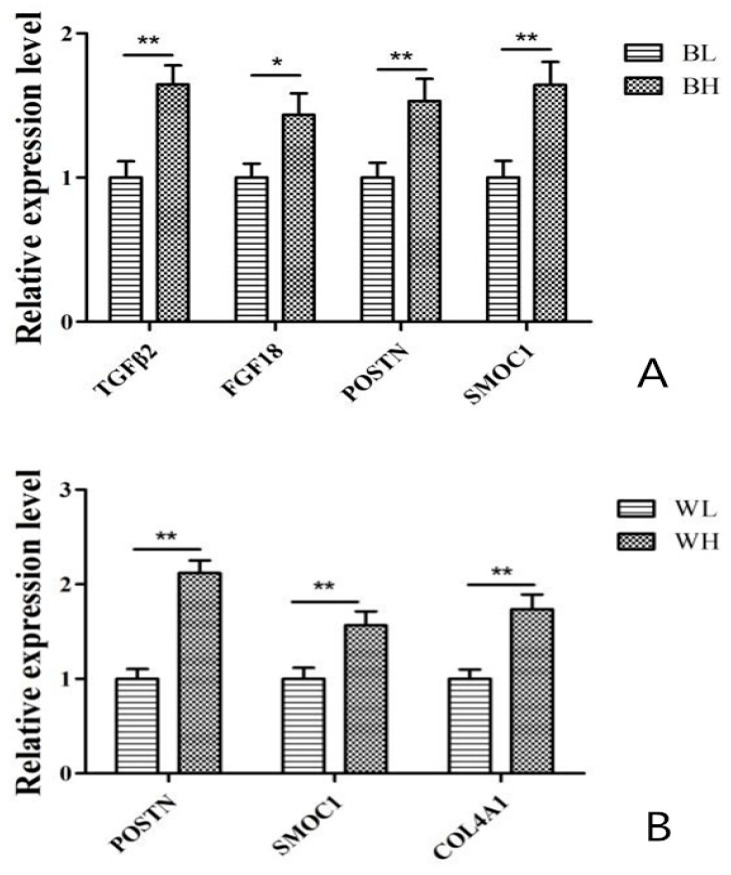
qPCR validation of DEGs in (**A**) BL-vs-BH and in (**B**) WL-vs-WH. The results were expressed as mean ± SD, * for *p* < 0.05, ** for *p* < 0.01.

In order to verify the expression levels of DEGs observed in our RNA-seq analysis, 4 randomly selected transcripts in BL-vs-BH (*TGFβ*2, *FGF*18, *POSTN* and *SMOC*1) and 3 randomly selected transcripts WL-vs-WH (*POSTN*, *SMOC*1 and *COL4A*1) shown to be differentially expressed on the basis of RPKM values were validated by qPCR. The qPCR results showed the similar regulated trend in the expression of these genes. Therefore, these results indicated the high reliability and accuracy of the RNA-seq data (Figure 6).

## 4. Discussion

Ovarian development and follicle formation in poultry is a dynamic and complex process, including follicle growth and selection, granulosa cell proliferation and differentiation and so forth. It is very important to understand the gene expression patterns in the ovary to improve the egg production rate in poultry [25]. In this study, the gene expression mechanisms and signal pathways in the ovaries of high- and low-yield Muscovy ducks were investigated by using RNA-seq technology. A total of 555,951,128 clean reads (46,329,260 on average) were discovered and the Mapped reads and Unique mapped reads were more than 60.28% and 57.81%, respectively. Besides, more than 16,276 genes were detected in each sample.

### 4.1. Analysis of DEGs

According to the DEGs in black Muscovy duck with high- and low-laying, several genes that may affect Muscovy duck egg production were identified, such as *TGFβ*2, *NGFR*, *CEBPD*, *CPEB*2, *POSTN*, *SMOC*1, *FGF*18, *EFNA*5 and *SDC*4. Moreover, some studies have shown that these regulatory transcription factors interacted with each other in regulating animal reproduction.

Transforming growth factor-β (TGF-β), one of the large family of multifunctional growth factors, regulates a wide range of biological activities related to morphogenesis, development and differentiation. There is a wide range of biological effects in TGF-β isoforms, including cell differentiation, cell proliferation, cell growth, extracellular matrix formation and immune function [26]. Some studies have found that the *TGFβ*2 gene may be involved in reproduction, such as Hu sheep [27]. Before maturation of porcine oocytes, the expression of *TGFβ*2 in large follicles was greater than that in small and medium follicles [28]. There is evidence that the up regulation of *TGFβ*2 may induce apoptosis during the regression of postovulatory follicle in chickens [29]. Ma et al. (2002) found that *TGFβ*2 was preferentially expressed in the ovaries and testes of chicken embryos, which may play a key role in regulating the development of germ cells of ovaries and testes in chicken embryos [30]. Nerve growth factor receptor (NGFR) is a multifunctional cell surface receptor, which widely exists in many cell types. The gene induces apoptosis and participates in injury, nervous system development and regeneration [31]. *NGFR* is expressed in female reproductive organs and plays an important role in regulating the growth, development and function of reproductive organs [32]. Studies have shown that *NGFR* was expressed in the follicles with different sizes in porcine ovaries during estrus, which may have an important impact on the ovarian function of sows [33]. In addition, *NGFR* is also expressed in the granulosa cells, thecal cells, stromal cells and luteal cells of goat ovarian [34]. The transcription factor C/EBPδ (CEBPD) is a member of the basic leucine zipper family transcription factor, which is related to cell proliferation, differentiation, growth arrest and apoptosis [35]. Becker et al. (2011) found that *CEBPD* had a higher proliferation rate in calf endometrium under the influence of steroid hormones [36]. All these studies have identified that the DEGs have important functions in the ovary. In this work, *TGFβ*2, *NGFR* and *CEBPD* were significantly expressed in high-laying Muscovy ducks, indicating that they may be very important for ovarian development in Muscovy ducks.

The cytoplasmic polyadenylation element binding protein family (CPEB), a sequence-specific RNA binding protein, regulates the physiological processes of germ cell development, cell division and cell differentiation by regulating the translation process [37,38]. The *CPEB*2 gene belongs to the CEPB family and has been discovered in mouse germ cells [39]. Its function is to promote meiotic maturation and embryonic development of porcine oocytes [40]. Periostin (POSTN) is a 90-kDa adhesion molecule that is secreted in the extracellular matrix. POSTN binds to cells via the Integrin/FAK/AKT signaling pathway and promotes cell migration, adhesion and survival of various cell types [41,42]. POSTN is associated with follicular granulosa cell proliferation [43,44]. POSTN affects the protein in the follicle and the insufficient protein in the follicular fluid may lead to unique characteristics of follicle development in lambs [45]. SPARC-related modular calcium binding 1 (SMOC1), one of the extracellular glycoproteins, is widely expressed in various tissues with localization to basement membranes. *SMOC1* may mediate intercellular signaling and cell type-specific differentiation during gonadal and reproductive tract development of fetal [46]. In this study, the differential expression of *CEPB*2, *POSTN* and *SMOC*1, which was in the ovarian tissues of high- and low-laying black and white Muscovy ducks, may have an important regulatory effect on the egg-laying mechanism of Muscovy ducks.

FGF18, a member of the FGF family, participates in the development of ovarian follicles [47]. FGF18 promotes the mitosis of thecal cells and apoptosis of bovine granulosa cells [48,49] and plays an important role in the regulation of steroid production in fetal bovine ovary [50]. Besides, Zhong et al. (2006) found that *FGF*18 gene was also presented in mouse thecal cells and oocytes [51]. Ephrin-A5 (EFNA5) is a member of the Ephrin-Eph family, which is highly expressed in granule cells [52]. EFNA5 has pivotal roles in cellular processes such as proliferation, survival, cell migration and invasion. Besides, EFNA5 also mediates cell apoptosis, proliferation and steroid production during follicular development in female mice [53]. In our study, *FGF*18 and *EFNA*5 were mainly expressed in the ovarian tissue of high-yield black Muscovy ducks, indicating that *FGF*18 and *EFNA*5 may play a key role in ovarian granulosa cell apoptosis and follicular development.

Syndecan4 (SDC4) is a cell surface heparan sulfate proteoglycan, which participates in cell adhesion [54,55] and is widely expressed in most adult tissues [56]. Studies have shown that the expression of *SDC*4 is related to follicular atresia in mice [57]. In addition, compared with the dominant follicles of cattle, the upregulation of *SDC*4 gene in ovulatory follicles may affect the ovulation promoting or luteinizing process [58]. In this study, the expression of *SDC*4 in the ovarian tissue of high-laying white Muscovy ducks was significantly up-regulated, which may be related to the high-egg production of Muscovy ducks.

### 4.2. Analysis of GO and KEGG

To better understand the possible functions involved in ovarian development in Muscovy ducks, the DEGs from each comparison group were annotated with the GO database. the results indicated that the DEGs played an important role in regulating duck ovarian development. Functional classification and pathway assignments were based on a KEGG analysis. In this study, 2 major pathways related to ovarian development were identified, namely PI3K-Akt signaling pathway and ovarian steroidogenesis. The PI3K/Akt pathway is an intracellular signaling pathway of great importance in the cell cycle process. Its plays an important role in regulating metabolism and has been widely investigated in cell proliferation, cell transformation, paracrine function and angiogenesis [59]. In the PI3K/Akt signal transduction pathway, with the help of phosphatidylinositol dependent kinase, protein kinase B (Akt) binds to cell membrane and exert biological effects [60]. Phosphatidylinositol-4,5-diphosphate 3-kinase (PI3K) is activated by many genes and it has been shown to be closely related to follicular recruitment and ovulation, selection and development of dominant follicles in mammalian ovaries [61,62,63,64].

Ovarian cell proliferation and apoptosis are important factors affecting ovarian function. Recent studies have shown that the follicular development and granulosa cell survival and proliferation can be improved by regulating the expression of PI3K/Akt signaling pathway [65]. Mishra et al. (2020) found that DEGs associated with high rates of egg production regulate PI3K-Akt signaling pathway at hypothalamic-pituitary-ovarian axis in laying hens [66]. The ovaries with reproductive activity are composed of small pre-hierarchical and maturing preovulatory follicles showing a hierarchy according to their size [67]. In addition, ovarian function is regulated by gonadotropins, including follicle stimulating hormone (FSH), luteinizing hormone (LH) and ovarian steroids. In animals, steroid hormones are synthesized from cholesterol by LH responsive theca cells and then converted into estrogens [68]. With the maturation of follicles, the concentration of steroid hormones such as estrogen and progesterone increases to exert their reproductive functions. The pathways in this study strongly indicated that PI3K-Akt signaling pathway and ovarian steroidogenesis played an important role in follicle development and productivity of Muscovy ducks.

Finally, we validated the RNA-seq results using qPCR to measure the expression of five DEGs (*TGFβ*2, *FGF*18, *POSTN*, *SMOC*1 and *COL4A*1), which showed that the results were reliable. In these five DEGs, the expression of *POSTN* and *SMOC*1 were high significantly different in BL-vs-BH and WL-vs-WH, indicating that *POSTN* and *SMOC*1 played an important role in regulating ovarian development of Muscovy ducks. Besides, the expression of *TGFβ*2 and *FGF*18 in BH were significantly or extremely significantly higher than that in BL and the expression of *COL4A*1 in WH were extremely significantly higher than that in WL, respectively. These results suggested that *TGFβ*2, *FGF*18 and *COL4A*1 may have relatively more important function in the regulation of egg production in Muscovy ducks.

## 5. Conclusions

In this study, several genes that may be important candidate genes involved in Muscovy duck ovarian development, such as *TGFβ*2, *NGFR*, *CEBPD*, *CPEB*2, *POSTN*, *SMOC*1, *FGF*18, *EFNA*5 and *SDC*4, were discussed. Go term annotation and KEGG pathway enrichment analysis showed that a number of significantly enriched DEGs may be related to PI3K-Akt signaling pathway and ovarian steroidogenesis in ovaries of Muscovy ducks. These data provide comprehensive gene expression information at the transcriptional level and will certainly accelerate the study of Muscovy duck ovarian development.

## Figures and Tables

**Table 1:** The feed composition for black Muscovy ducks.

Ingredient	Content (%)	Nutrient	Content (%)
Corn	56.00	Crude protein	15.700
Soybean meal	23.80	Calcium	0.900
Corn gluten meal	10.00	Total phosphorus	0.680
Limestone	7.00	Available phosphorus	0.450
CaHPO_4_	1.50	Salt	0.370
Premix	1.00	Lysine	0.760
NaCl	0.30	Methionine	0.387
Lys·HCl	0.30	Methionine + Cystine	0.654
DL-Met	0.10	Isoleucine	0.534
Total	100.00	Threonine	0.579
		Tryptophan	0.194
		Crude fiber	4.100
		Crude fat	3.400
		Crude ash	5.200
		Avian metabolizable energy	2875 Mcal·kg^−1^

**Table 2:** qPCR primers of Muscovy duck.

Primer	Sequences(5′–3′)	GeneBank Accession Number	Product Length (bp)
*POSTN*	F:AACACGCTTGAAGTTGGC R:TCAATGAGGTGGATAACG	XM_005018343.4	107
*SMOC*1	F:CCGCTTCAGACCTGGCAATC R:CTCAAGAGACAGGCCCAGTTTCTAC	XM_013101462.3	149
*TGFβ*2	F:ATCTACAACAGCACCAGGGAC R:TAGCTTGGTGGGATGGCA	XM_027453142.1	155
*FGF*18	F: ATGTTTGTTGCCGAGGAG R:TGTTTCCCGCTTGTCCTG	XM_027468582.1	125
*COL4A*1	F:GGAGAAATGGGAGTTATGGG R:TTGGCCTTTGAGACTAACC	XM_027444310.1	213
β-actin	F:TATGTCGCCCTGGATTTCG R:CTCAAGAGACAGGCCCAGTTTCTAC	XM_013101462.3	162

**Table 3:** Summary of reads and matches.

Reads	Clean Reads	Mapped Reads	Unique Mapped Reads	Detected Genes
BH1	46,357,720	63.07%	60.66%	17,786
BH2	46,947,348	63.87%	61.37%	17,822
BH3	46,070,474	62.75%	60.33%	17,699
BL1	47,534,374	67.40%	64.36%	16,847
BL2	47,067,606	64.27%	61.86%	17,926
BL3	45,252,048	63.94%	60.66%	16,276
WH1	46,942,582	64.33%	61.76%	17,371
WH2	44,667,726	61.96%	59.67%	17,553
WH3	47,963,328	63.71%	61.09%	17,549
WL1	46,411,962	65.58%	62.91%	17,553
WL2	46,391,890	60.28%	57.81%	17,417
WL3	44,344,070	64.77%	61.92%	17,286

## Data Availability

The datasets generated for this study can be found in the NCBI SRA (Submission: SUB7071226). Bioproject #PRJNA641492 and Biosamples #SAMN14252184-SAMN14252193.

## Supplementary Materials

The following are available online at https://www.mdpi.com/2073-4425/12/1/57/s1, Figure S1: The Pearson correlation coefficient between samples, Table S1: Sample quality control information
