# Supplementary material for: Comparative Transcriptome Profiling of Ovary Tissue between Black Muscovy Duck and White Muscovy Duck with High- and Low-Egg Production
Source: Genes (Basel). 2020 Dec 31;12(1):57. doi: 10.3390/genes12010057 (PMC7824526; doi:10.3390/genes12010057)
Supplement: Supplementary file 1 [file genes-12-00057-s001.zip › Table S1.pdf]

Table S1. Sample quality control information

Sample	Concentration (ng/μL)	OD260/230	OD260/280	RIN
BH1	1,098.3	2.03	1.88	8.2
BH2	1,195.5	2.05	1.91	9.9
BH3	1,143.0	2.08	1.96	8.8
BL1	1,010.1	2.14	2.01	7.8
BL2	1,533.5	2.09	2.07	8.4
BL3	953.9	2.03	2.10	8.2
WH1	1,340.2	2.07	1.98	8.5
WH2	1,215.2	2.10	1.88	7.9
WH3	1,038.6	2.12	1.94	8.0
WL1	875.6	2.10	1.97	8.6
WL2	1,154.1	2.08	2.00	8.5
WL3	1,085.3	2.04	1.94	8.1
