# Supplementary figures and images for: Comparative Transcriptome Profiling of Ovary Tissue between Black Muscovy Duck and White Muscovy Duck with High- and Low-Egg Production
Source: Genes (Basel). 2020 Dec 31;12(1):57. doi: 10.3390/genes12010057 (PMC7824526; doi:10.3390/genes12010057)

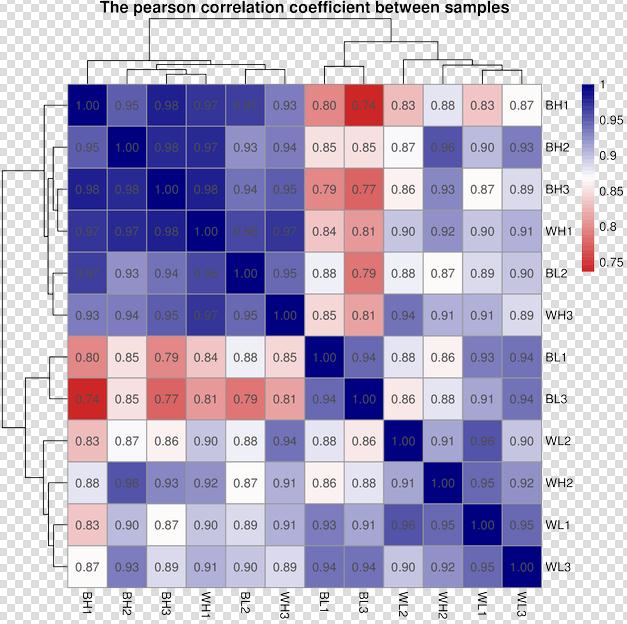

Supplement: Supplementary file 1 [file genes-12-00057-s001.zip › Figure S1. The Pearson correlation coefficient between samples..jpg]
